# Clinical efficacy of enhanced recovery after surgery (ERAS) program in patients undergoing radical prostatectomy: a systematic review and meta-analysis

**DOI:** 10.1186/s12957-020-01897-6

**Published:** 2020-06-17

**Authors:** Yurong Zhao, Shaobo Zhang, Bianjiang Liu, Jie Li, Hanxia Hong

**Affiliations:** Department of Urology, The First Affiliated Hospital of Nanjing Medical University, Nanjing, 210029 China

**Keywords:** Enhanced recovery after surgery, Radical prostatectomy, Meta-analysis, Systematic review

## Abstract

**Background:**

Enhanced recovery after surgery (ERAS) protocol has been identified to be beneficial in the amount of operations such as gastrointestinal surgery. However, the efficacy and safety in robot-assisted laparoscopic prostatectomy/laparoscopic radical prostatectomy (RALP/LRP) still remain controversial.

**Method:**

We searched randomized controlled trials and retrospective cohort studies comparing ERAS versus conventional care for prostate cancer patients who have undergone RALP/LRP. ERAS-related data were extracted, and quality of included studies was assessed using the Newcastle-Ottawa quality assessment scale and the Jadad scale.

**Result:**

As a result, seven trials containing 784 prostate cancer patients were included. ERAS was observed to be significantly associated with shorter length of hospital stay (SMD − 2.55, 95%CI − 3.32 to − 1.78, *P* < 0.05), shorter time to flatus (SMD − 1.55, 95%CI − 2.26 to − 0.84, *P* < 0.05), shorter time to ambulate (SMD − 6.50, 95%CI − 10.91 to − 2.09, *P* < 0.05), shorter time to defecate (SMD − 2.80, 95%CI − 4.56 to − 1.04, *P* < 0.05), and shorter time to remove drainage tube (SMD − 2.72, 95%CI − 5.31 to − 0.12, *P* < 0.05). Otherwise, no significant difference was reported in other measurements.

**Conclusions:**

In conclusion, ERAS can reduce length of hospital stay, time to flatus, time to defecate, time to ambulate, and time to remove drainage tube in prostate cancer patients who have undergone RALP/LRP compared with conventional care.

## Introduction

Enhanced recovery after surgery (ERAS), firstly introduced by Danish Doctor Wilmore and Kehlet, consists of a series of evidence-based procedures for optimizing perioperative treatment [1]. ERAS was designed to reduce the length of hospital stay, relieve patients’ psychological stress response, and reduce perioperative complications [2].

Prostate cancer is the most common cancer of the genitourinary tract in men [3]. Sufficient evidences have shown that compared with open radical prostatectomy (ORP), robot-assisted laparoscopic prostatectomy/laparoscopic radical prostatectomy (RALP/LRP) is associated with lower blood loss and transfusion rate, as well as less hospitalization duration [4–6]. However, when it comes to the postoperative complications and functional outcome, no significant difference was reported between RALP/LRP and ORP [4, 5]. Until now, there was still the lack of well-recognized study exploring the clinical efficacy and safety of the ERAS program in RALP/LRP, which hindered the wide application of ERAS program in patients with prostate cancer.

Nowadays, ERAS has been applied in various aspects such as gastrointestinal surgery and gynecological surgery and promoted its advantages. In this study, we are aimed to perform a systematic review and meta-analysis to qualitatively and quantitatively compare the EARS program with conventional care (TC) to evaluate the clinical efficacy and safety of ERAS program for prostate cancer patients who have undergone RALP/LRP.

## Methods

### Literature search

This systematic review and meta-analysis was strictly guided by the PRISMA principles, and the checklist of PRISMA was presented in Supplemental Table 1. Literature review was comprehensively carried out in the following databases: Medline (via PubMed), Embase, the Cochrane Central Register of Controlled Trials (Cochrane Library), WANFANG, and CNKI databases, to screen published articles reporting the outcomes of ERAS program application in the RP patients. Following Medical Subject Headings (MeSH) search terms were used: [“fast-track surgery” OR “fast-track rehabilitation” OR “enhanced recovery protocol” OR “enhanced recovery after surgery”] AND [“prostatic Neoplasms” OR “prostate tumor” OR “prostatic cancer” OR “prostatectomy” OR “radical prostatectomy”]. To sort out any study which might have been missed, we checked reference lists of all related articles and published abstracts from authoritative academic conferences.

### Inclusion and exclusion criteria

Eligible studies were identified according to following inclusion criteria: (1) studies which was designed to compare the group treated with ERAS program with TC program in patients who underwent RALP/LRP operations; (2) a clear and comprehensive ERAS protocol should be provided in the study design; and (3) at least three of the following parameters should be included in the study design: the average length of hospital stay, duration of flatus or defecation, and rates of complications, readmissions, or mortality. Moreover, the exclusion criteria was listed below: (1) studies published in other than English or China; (2) there was the lack of available information extracted or insufficient data for pooling results from the studies; (3) review or case report; and (4) studies based on non-human research. Two independent authors (YR Zhao and BJ Liu) screened all searched studies for final pooling analysis, whereas discrepancies were resolved by consensus.

### Data extraction and quality assessment

Two independent authors reviewed all eligible studies in this systematic review and meta-analysis and extracted the following outcomes of interest: (1) name of first author, nationality, race, gender proportion, average age, publication year, and body mass index (BMI); (2) numbers of ERAS and TC group, prostate-specific antigen (PSA) in nanograms per milliliter; and (3) ERAS program-relevant characteristics, including operative time, blood loss, length of hospital stay, urinary tract infection, time to ambulate, time to defecate, time to remove urethral catheter, time to flatus, deep venous thrombosis, time to regular diet, time to remove drainage tube, nausea, intestinal obstruction, and urinary leakage.

The methodological quality of randomized controlled trials (RCTs) was assessed by the Jadad scale, including randomization, blinding, and dropouts or withdrawals [7]. Otherwise, the Newcastle-Ottawa quality assessment scale (NOS) was used to score the quality of retrospective studies by three domains (selection, comparability, and exposure) [8].

### Statistical analysis

The pooled data was calculated to evaluate the strength of the difference between the ERAS program and the TC program by standardized mean difference (SMD) for continuous variables and odds ratio (OR) for binary subjects with 95% confidence intervals (95% CIs). A *P* value < 0.05 was considered to be of statistical significance. Statistical heterogeneity was determined by the *I*^*2*^, which was defined as 100% × (*Q* − df)/*Q*, where *Q* is Cochran’s heterogeneity statistic, and df is the degrees of freedom. A fixed-effects model was used when the *I*^*2*^ value was ≤ 25%, whereas a random-effects model would be selected [9]. Only prospective studies were included for sensitivity analyses. Egger’s regression test was used to explore the potential publication bias among all eligible studies. All statistical analyses were performed in the STATA software (StataCrop, release 15.1, College Station, TX, USA).

## Results

### Basic characteristics of eligible studies

**Fig. 1:**
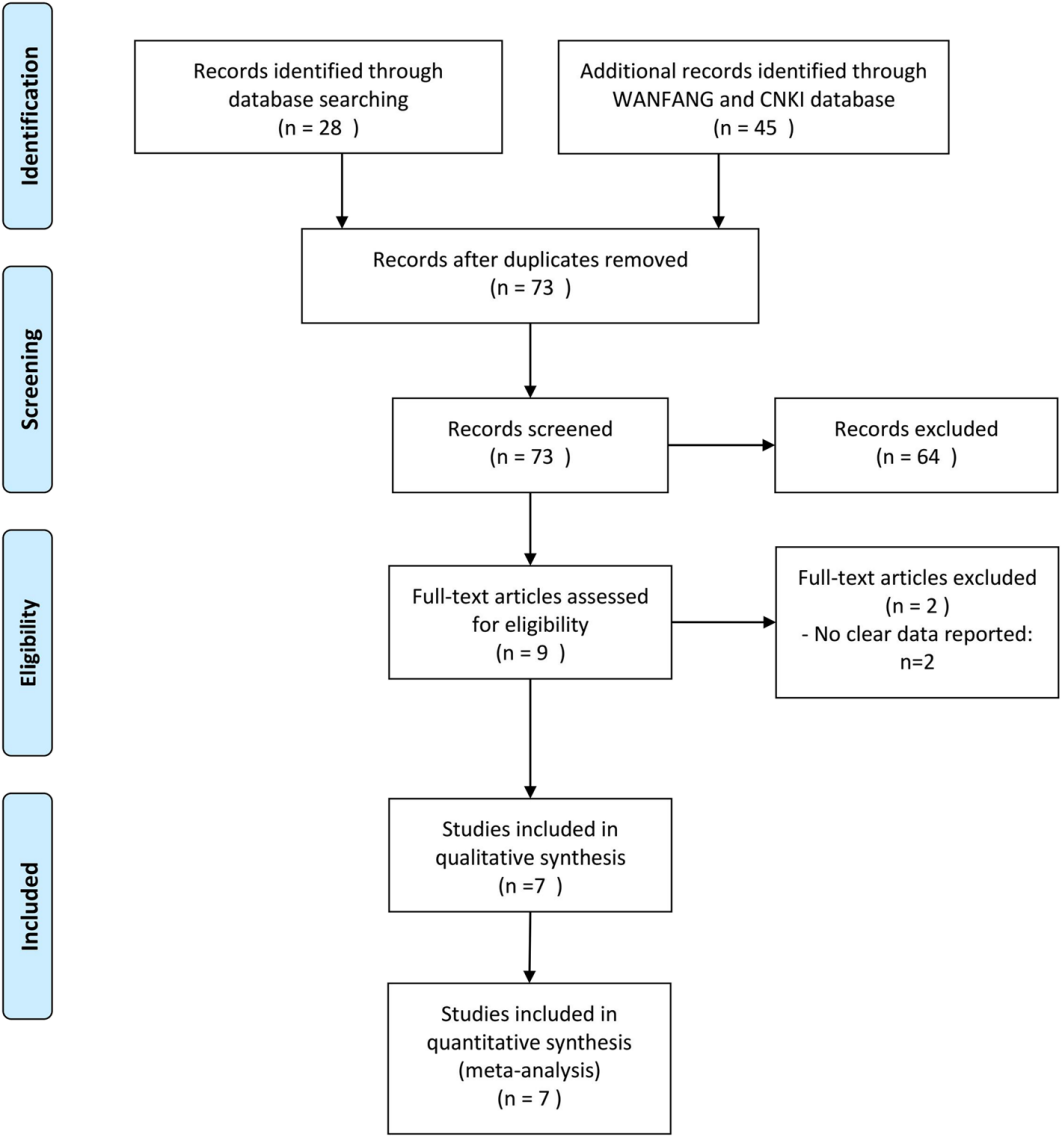
Flow diagram of study selection in the systematic review and meta-analysis

The flow diagram of selection of eligible studies is presented in Fig. 1. Seven trials [10–16], including five RCTs [12–16] and two retrospective cohort studies [10, 11], were eligible for systematic review and meta-analysis. In a total, all included studies evaluated 784 cases of prostate cancer patients, of which 379 subjects undergoing ERAS management whereas 405 undergoing conventional perioperative management. Mean age of enrolled subjects ranged from 62.8 to 70.9 years in ERAS group and 61.9 to 70.0 years in the TC group. The mean PSA value ranged from 7.2 to 44.5 ng/ml in ERAS group and 10.3 to 31.35 ng/ml in the TC group. The basic characteristics of the included studies are shown in Table 1. The ERAS program technical measures reported in all studies can be approached in Supplementary Table 1.

**Table 1:** Basic characteristics of included studies in the systematic review

Author	Country	Design	ERAS group/control group	Operation	Age (year)		PSA (ng/ml)		BMI	
					ERAS group	Control group	ERAS group	Control group	ERAS group	Control group
**Magheli et al.** [12]	Germany	RCT	25/25	LRP	61.8 ± 4.7	61.9 ± 7	7.2 ± 4.9	10.3 ± 8.6	/	/
**Ren et al.** [15]	China	RCT	9/9	LRP	69.2		12		/	/
**Huang et al.** [10]	China	Retrospective cohort study	36/37	RALP	62.1 ± 6.9	63.5 ± 7.4	13.44 ± 8.01	15.4 ± 10.59	23.1 ± 2.1	23.5 ± 2.2
**Pan and Li** [14]	China	RCT	50/50	LRP	69.3 ± 7.32		/	/	/	/
**Yu and Wang** [16]	China	RCT	26/25	RALP	67.65 ± 7.37	72.00 ± 6.07	31.55 ± 22.57	31.35 ± 31.46	21.88 ± 2.49	20.84 ± 3.15
**Dong et al.** [13]	China	RCT	109/95	RALP/LRP	66.76 ± 5.83	66.95 ± 5.70	/	/	22.39±1.47	22.32 ± 1.54
**Lin et al.** [11]	China	Retrospective cohort study	124/164	LRP	70.9 ± 3.6	70 ± 4.3	44.5 ± 22.3	36.8 ± 23.2	20.3 ± 1.5	20.4 ± 1.4

*RCT* randomized controlled trial, *LRP* laparoscopic radical prostatectomy, *RALP* robot-assisted laparoscopic prostatectomy, *PSA* prostate specific antigen, *BMI* body mass index

Using the Jadad scale, three of all five RCTs [12, 15, 16] scored three points, representing medium study quality; two [13, 14] scored four points, which suggested high quality. Both of eligible retrospective cohort studies scored six to seven points by the NOS scale, and medium study quality was identified (Table 2).

**Table 2:** Results of elements evaluated in each enhanced recovery after surgery (ERAS) protocol

Study	Huaxiang Yu	Nannan Dong	Jie Pan	Jian Ren	Zhichao Huang	Chunhua Lin	Ahmed Magheli
	2018	2018	2018	2014	2018	2019	2011
Preoperative education	YES	YES	YES	NG	YES	YES	NG
Mechanical bowel preparation omission	YES	YES	YES	YES	YES	YES	YES
Limited preoperative fast	YES	YES	YES	NG	YES	YES	YES
Preoperative carbohydrate loading	YES	NG	YES	NG	YES	NG	NG
Preoperative nutrition	NG	NG	YES	NG	NG	NG	YES
Venous thromboembolism prophylaxis	YES	YES	YES	YES	YES	YES	YES
Epidural analgesia	YES	NG	NG	YES	NG	YES	NG
Prevention of intraoperative hypothermia	YES	NG	NG	NG	NG	YES	NG
Goal-directed fluid therapy	YES	NG	NG	NG	YES	YES	NG
Avoidance of nasogastric intubation	YES	NG	YES	NG	NG	YES	NG
Prevention of paralyticileus	YES	NG	YES	YES	YES	NG	NG
Pain control	YES	YES	YES	YES	YES	YES	YES
Early mobilization	YES	YES	YES	YES	YES	YES	YES
Early oral diet	YES	YES	YES	YES	YES	YES	NG
Non-opiate oral analgesia	YES	NG	YES	YES	YES	YES	NG
Total elements	14	7	12	8	11	12	6

*NG* not given

### Meta-analysis results

#### Length of hospital stay (LOS)

**Fig. 2:**
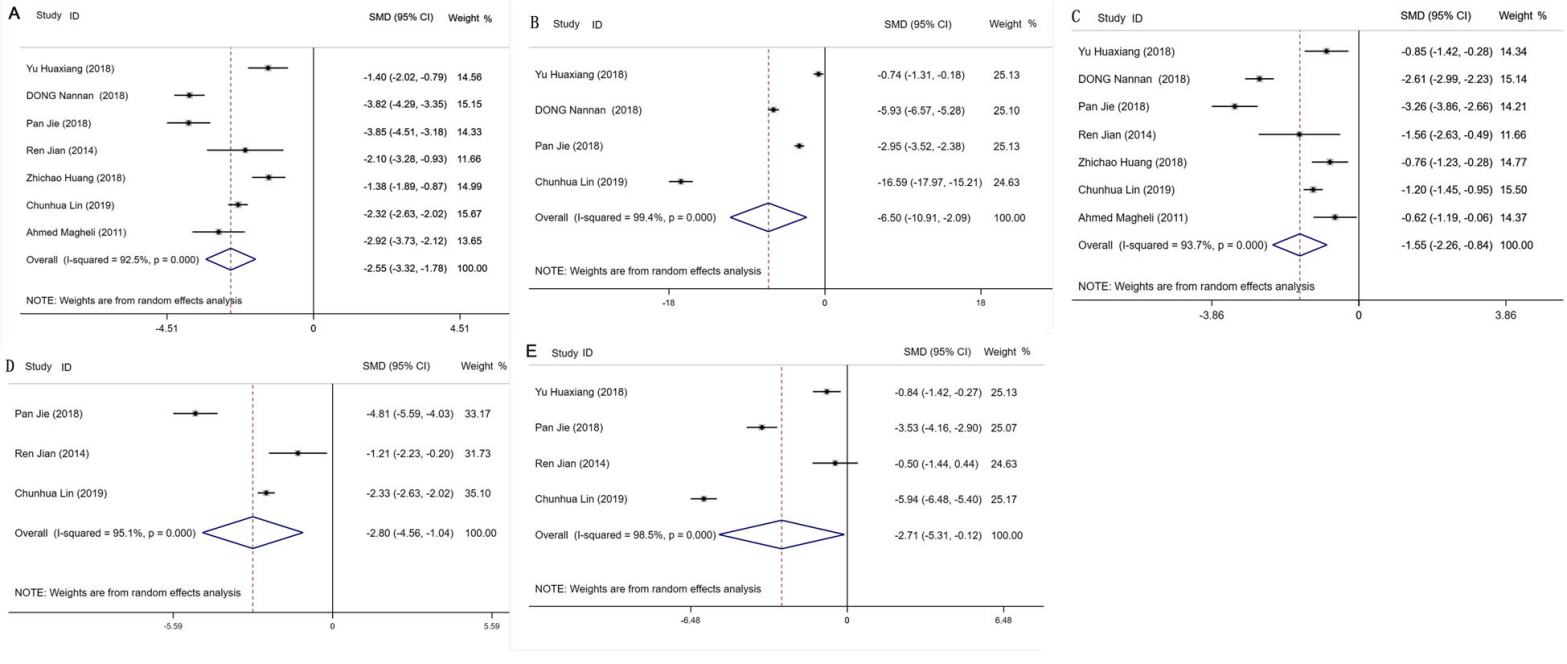
Results of meta-analysis for enhanced recovery after surgery (ERAS) in patients undergoing radical prostatectomy, including the length of hospital stay (**a**), time to ambulate (**b**), time to flatus (**c**), time to defecate (**d**), and time to remove drainage tubes (**e**)

Seven studies [10–16] including 784 participants reported length of hospital stay (LOS). We observed a statistically significant shorter LOS in ERAS group compared with TC group (SMD − 2.55, 95%CI − 3.32 to − 1.78, *P* < 0.001; Fig. 2a).

#### Time to ambulate

Four studies [11, 13, 14, 16] including 639 participants addressed attention on the time to ambulate. The time to ambulate in ERAS group is statistically shorter (SMD − 6.50, 95%CI − 10.91 to − 2.09, *P* = 0.004; Fig. 2b).

#### Time to flatus

Seven studies [10–16] including 784 participants reported time to flatus. In a random-effects model, the result indicates a statistically significant shorter time to flatus in ERAS group (SMD − 1.55, 95%CI − 2.26 to − 0.84, *P* < 0.001; Fig. 2c).

#### Time to defecate

Three studies [11, 14, 15] reported the time to defecate including 406 participants. The result indicates a statistically significant shorter time to defecate in ERAS group (SMD − 2.80, 95%CI − 4.56 to − 1.04, *P* = 0.002; Fig. 2d).

#### Time to remove drainage tubes

Four studies [11, 14–16] reported the time to remove drainage tubes including 457 participants. The result indicates a shorter drainage tube removal time (SMD − 2.71, 95%CI − 5.31 to − 0.12, *P* = 0.041; Fig. 2e).

#### Other parameters

The other parameters including operative time, blood loss, time to remove ureteral catheter, time to regular diet in days, and rate of postoperative complications (nausea, intestinal obstruction, urinary tract infection, urinary leakage, deep venous thrombosis) were presented to have no statistical significance (Supplemental Figure 1 and 2). Moreover, no publication bias was observed by the Egger’s test in the meta-analysis of each parameter (*P* > 0.05).

## Discussion

In the present study, we included seven eligible studies to explore the efficacy and safety of the ERAS program in patients who underwent RP surgery and reported that the ERAS program resulted in significantly shorter length of hospital stay and reduced time to flatus, defecate, ambulate, and remove drainage tubes. However, the differences of interpretative measures (including operative time and blood loss) and the occurrence of complications (including nausea, intestinal obstruction, urinary tract infection, urinary leakage, and deep venous thrombosis) remained no statistical significance.

To date, numerous articles have been performed to explore the efficacy and safety of ERAS program in laparoscopic surgery of various diseases, such as colorectal cancer, gastric cancer, bladder cancer, and hepatocellular cancer [17–21]. Ni et al. [17] reported 13 RCTs of ERAS program in laparoscopic colorectal cancer surgery. Compared with patients in TC group, patients in ERAS group have the shorter time to leave hospital and recover gastrointestinal function and lower postoperative complication rates. Specifically, IL-6 and CRP levels of patients in ERAS group are proved to be lower. Wee et al. [18] reported 23 studies (including 14 RCTs) of ERAS program in gastric cancer surgery in 2018 and demonstrated that ERAS program in gastric cancer surgery can reduce hospital stay, costs, surgical stress response, and time to return of gut function as compared to conventional care. Xiao et al. [19] reported 16 trails (including 8 retrospective and 8 prospective trials) of ERAS program in bladder cancer surgery in 2019 and showed that ERAS protocols are associated with a faster return of bowel function, reduced incidence of POI, and shorter LOS when compared to SC in patients undergoing RC. Consistent with above studies, we observed that ERAS program showed significantly shorter time to first flatus and the length of hospital stay in patients with prostate cancer. On the other hand, the postoperative complications rate remains controversial among these studies. In this meta-analysis, the difference of postoperative complications rate between ERAS group and TC group has no statistical significance, and several studies shared the same result with us [21, 22]. The other four studies [17, 19, 20, 23] shared the result that ERAS group has a lower postoperative complications rate. Thus, more studies elevating the operative compliment rates have to be done. Particularly, two measurements, including shorter time to remove the drainage tube and first ambulate, were demonstrated to be statistically significant in our analysis, which was not explored in these three studies. Moreover, four of our included studies all reported that ERAS program can reduce the hospitalization cost. Although our study got the similar result, we considered the result had no clinical significance, considering the cost of RLP and RALP was various.

There are limitations which cannot be ignored in our study. The major limitation of this review is the number of studies we included; we only included seven studies.

## Conclusion

ERAS program can significantly reduce the length of hospital stay and the time to ambulate, defecate, and flatus in patients undergoing the RALP/LRP, which could be recognized as great clinically efficacy and safety. However, our results should be interpreted with great caution due to some limitations. A large-scale, well-designed, multi-center RCT should be conducted to confirm our results.

## Supplementary information

**Additional file 1: Supplemental Figure 1.** Results of meta-analysis for enhanced recovery after surgery (ERAS) in patients undergoing radical prostatectomy, including operative time (A), blood loss (B), time to regular diet (C) and time to remove ureteral catheter (D).

**Additional file 2: Supplemental Figure 2.** Results of meta-analysis for enhanced recovery after surgery (ERAS) in patients undergoing radical prostatectomy, including complications of urinary tract infection (A), deep vein thrombosis (B) and nausea (C).

**Additional file 3: Supplemental Table 1.** The PRISMA checklist.

## Data Availability

The current study was based on the results of relevant published studies.
