# Supplementary figures and images for: Clinical efficacy of enhanced recovery after surgery (ERAS) program in patients undergoing radical prostatectomy: a systematic review and meta-analysis
Source: World J Surg Oncol. 2020 Jun 17;18:131. doi: 10.1186/s12957-020-01897-6 (PMC7301489; doi:10.1186/s12957-020-01897-6)

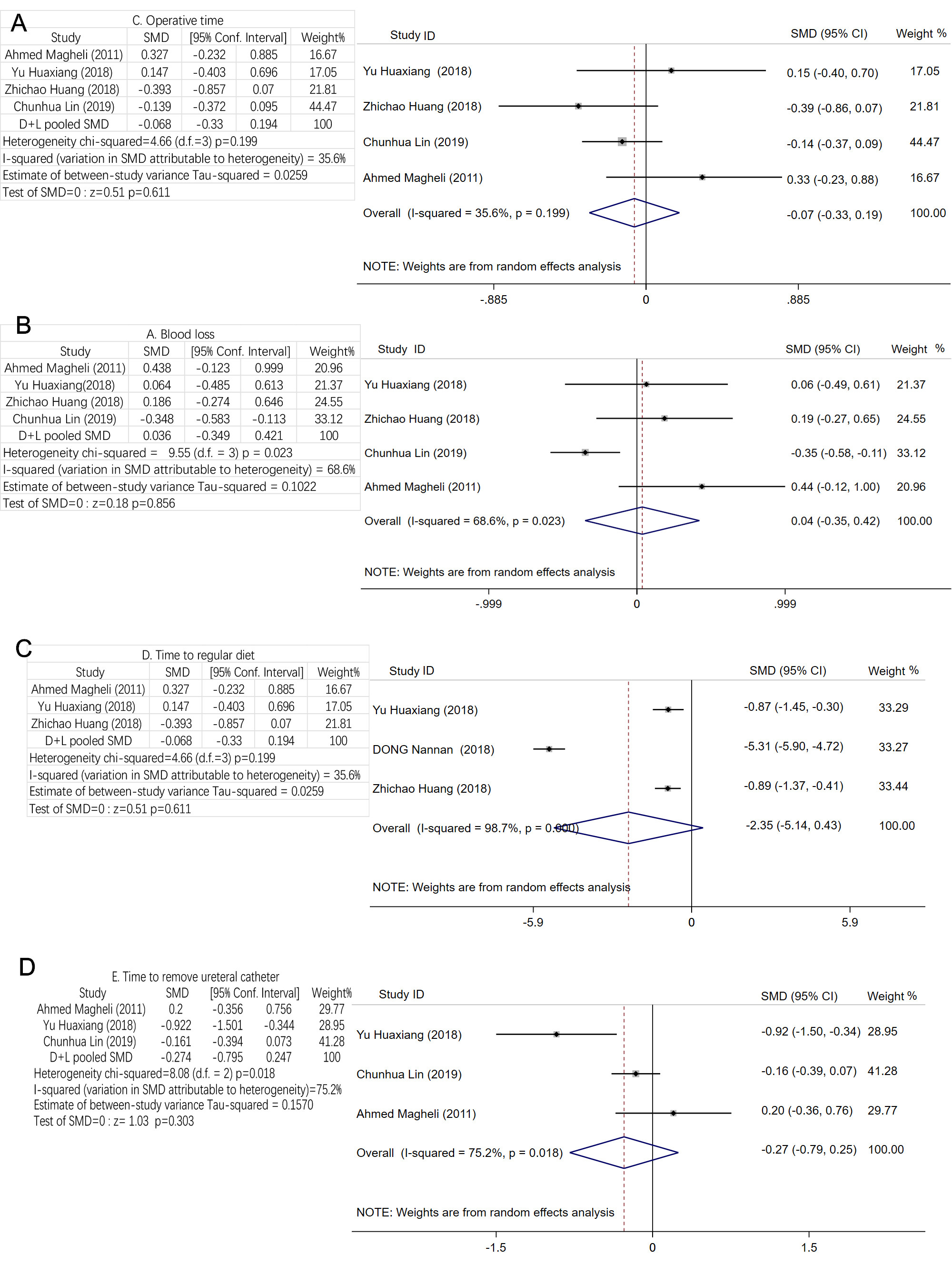

Supplement: Supplementary file 1 — Additional file 1: Supplemental Figure 1. Results of meta-analysis for enhanced recovery after surgery (ERAS) in patients undergoing radical prostatectomy, including operative time (A), blood loss (B), time to regular diet (C) and time to remove ureteral catheter (D). [file 12957_2020_1897_MOESM1_ESM.jpg]

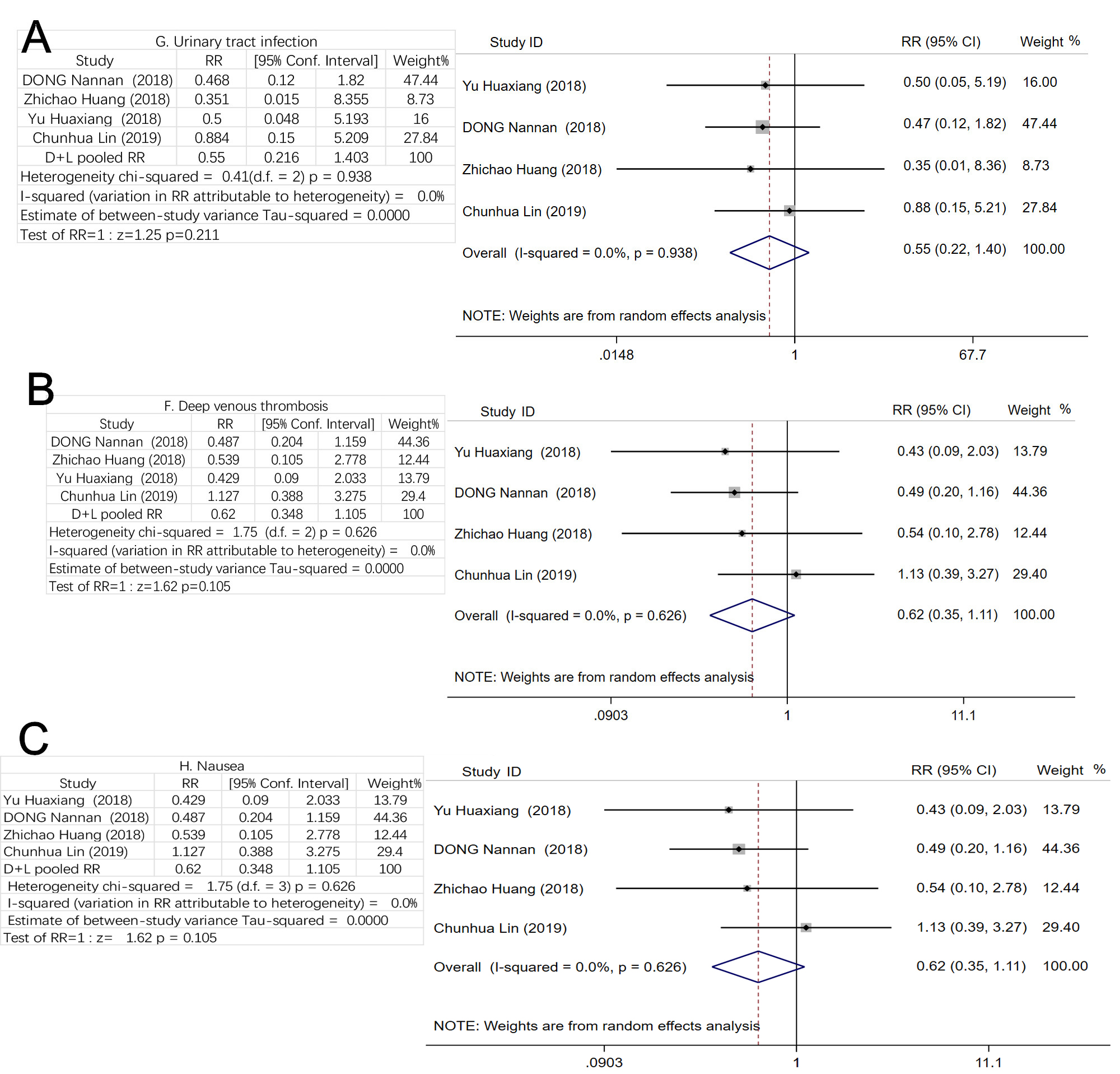

Supplement: Supplementary file 2 — Additional file 2: Supplemental Figure 2. Results of meta-analysis for enhanced recovery after surgery (ERAS) in patients undergoing radical prostatectomy, including complications of urinary tract infection (A), deep vein thrombosis (B) and nausea (C). [file 12957_2020_1897_MOESM2_ESM.jpg]
